# Automatic referrals within a cystic fibrosis multidisciplinary clinic improve patient evaluation and management

**DOI:** 10.1016/j.jcte.2021.100259

**Published:** 2021-06-12

**Authors:** Amy Darukhanavala, Ted Kremer

**Affiliations:** a Department of Pediatric Endocrinology, University of Massachusetts Medical Center, Worcester, MA, USA; b Department of Pediatric Pulmonology, University of Massachusetts Medical Center, Worcester, MA, USA

**Keywords:** Cystic fibrosis, Endocrinology, Multidisciplinary clinic, Referral

## Abstract

AUTOMATIC REFERRALS WITHIN A CYSTIC FIBROSIS MULTIDISCIPLINARY CLINIC IMPROVE PATIENT EVALUATION AND MANAGEMENT.

**Background:**

Cystic fibrosis (CF) affects multiple systems beyond the pulmonary system, including the gastrointestinal and endocrine systems. Many CF clinics focus on pulmonary effects, initiating referrals to other specialties only when a condition has been identified by the primary pulmonary team. Unfortunately, many extrapulmonary manifestations of cystic fibrosis may be overlooked. Thus, implementing a multidisciplinary clinic with automatic referrals to designated subspecialists may improve patient care.

**Methods:**

This retrospective review of medical records examined the effects of integrating a pediatric endocrinologist into the University of Massachusetts Memorial Medical Center Pediatric CF Clinic in March 2017. In this new CF/Endocrinology clinic, all patients scheduled to see a pulmonologist were automatically referred to pediatric endocrinology. We compared rates of referrals to pediatric endocrinology, oral glucose tolerance tests (OGTTs), and bone density (DEXA) scans before (2013–2016) and after (2017–2020) implementation of this clinic. We also recorded endocrine disorders being evaluated and/or treated after implementation.

**Results:**

The rate of referral to pediatric endocrinology increased from before (4%) to after (82%) (p < 0.0001) implementation of the CF/Endocrinology Clinic. OGTT and DEXA scan screening rates also increased from 7% to 65% (p < 0.0001) and from 6% to 63% (p = 0.0011), respectively. Before implementation, patients were evaluated by endocrinology primarily for CF-related diabetes. After implementation, the diversity of endocrine conditions under evaluation and/or management increased substantially; the most common were vitamin D insufficiency/deficiency (37.2% of clinic patients), glycemic dysregulation (36.8%), and poor weight gain/failure to thrive (17.5%).

**Conclusion:**

Implementing a multidisciplinary CF clinic with automatic referrals to pediatric endocrinology improves patient care by promoting early detection and management of endocrine concerns that may have been overlooked and by increasing OGTT and DEXA screening rates.

## Introduction

Cystic fibrosis (CF) is the most lethal autosomal recessive disorder in Caucasians, with a worldwide prevalence of 1/2500 live births. It caused by a mutation of the cystic fibrosis transmembrane conductance regulator (*CFTR*) gene. Although CF is most commonly considered a lung disease, *CFTR* is widely expressed throughout the body. As a result, multiple organ systems are affected, including the gastrointestinal and endocrine systems [1].

Many CF clinics focus predominantly on reducing pulmonary effects of this disease, initiating referrals to endocrinologists and gastroenterologists only when a corresponding condition has been identified. When following this approach, many subtle non-pulmonary concerns may be overlooked, potentially impacting overall care in a negative manner.

CF is a difficult chronic disease, which requires patients and their families to undergo multiple clinic visits, frequent hospitalizations for prolonged exacerbations, and daily arduous treatments. It imposes high physical and psychological costs for patients and their families, and efforts to reduce the burdens of this disease are necessary [2]. Clinical benefits of multidisciplinary clinics are well described in a variety of complex medical disorders. An integrated team approach is the preferred management approach, which is exceptionally valuable for optimally managing individuals with systemic disease [3]. A collaborative care team limits visits and exposure to hospitals and clinics, thereby reducing time and financial burdens and decreasing the mental and physical stress associated with multiple subspecialty visits. In addition, patients are pleased with the improved communication between providers and are more confident in their overall care [4], [5], [6], [7], [8], [9]. Numerous studies have shown that multidisciplinary clinics improve outcomes [10], [11], [12], reduce mortality [13], [14], and are a cost-effective approach to patient care [4], [5]. However, limited studies were identified which assess the utilization and efficacy of automatic referrals in a multidisciplinary CF clinic [15].

CF clinics without routine access to an endocrinologist may be less likely to screen for endocrine complications of CF. Endocrine disorders identified in CF patients include, but are not limited to, disorders of linear growth (20% of patients) [16], poor weight gain or failure to thrive (20%) [17], disorders of puberty (20%–30%) [18] , menstrual irregularities (50%) [19], vitamin D insufficiency or deficiency (90%) [1], bone disease (55%–65%) [20], hypogonadism (25%–30%) [16], adrenal insufficiency secondary to prolonged glucocorticoid use (8%) [21], glycemic dysregulation (18%–47%) [22], and CF- related diabetes (CFRD) (40%–50%) [23].

Although the importance of establishing a collaborative care model for CF is inherently understood by the CF medical community, data on how to implement such a model are limited. In March 2017, the University of Massachusetts (UMass) Medical Center Pediatric Pulmonary Division and Pediatric Endocrinology Division designed an integrated weekly clinic to further address the endocrine complications of patients with CF. Through this process, endocrinology referrals became automatic within our CF multidisciplinary clinic. This achieved our goals of improving screening, detection, and management of endocrine-related disorders in our pediatric CF population.

## Material and methods

### Establishing the CF/Endocrinology clinic

UMass Medical Center, which is located in Worcester, Massachusetts, serves central and western Massachusetts. Established in 1985, the UMass Memorial Cystic Fibrosis Center was accredited by the national Cystic Fibrosis Foundation in 1993. The center currently serves 125 patients with CF, with 55 seen specifically in the Pediatric CF Clinic (patients aged 0–25 years).

Since its inception, the Pediatric CF Clinic has expanded to offer a multidisciplinary approach to patient care by including healthcare providers, registered dieticians, respiratory therapists, pharmacists, a social worker, and a psychologist. A pediatric endocrinologist joined the CF team in March 2017 to address and manage the underlying endocrine disorders of CF, thereby establishing a CF/Endocrinology Clinic. In this paper, we provide a model of a successful multidisciplinary approach to improved care of the CF pediatric patient.

In March 2017, initial efforts to establish multidisciplinary care were aimed at incorporating pediatric endocrinology in routine CF monitoring and management. This was achieved through multiple means, including direct presence of the endocrinologist at the introductory clinic visits with patients, at the weekly CF division meetings and bi-monthly patient care rounds, and at CF informational and social events provided for patients and their families.

Patient education on the role of a pediatric endocrinologist in a CF clinic was the primary focus during the first 6 months of establishing the CF/Endocrinology Clinic. Introductory clinic meetings with the pediatric endocrinologist allowed for in-depth explanations of the possible endocrine disorders that occur with CF. These discussions highlighted the importance of monitoring for and evaluating and treating disorders of growth and puberty, bone disease and glucose dysregulation/CFRD. Families were introduced to these possible concerns and educated on their impact on CF morbidity and mortality. Presentations by the endocrinologist at CF social and educational events, such as the UMass CF Family Night, provided further information regarding the effects of CF on the endocrine system. These efforts helped promote heightened awareness and understanding of the importance of a multidisciplinary approach among CF patients and their families.

These initial efforts had the added benefit of identifying individual endocrine concerns from both the families and healthcare providers perspectives. As families became progressively more educated, previously unidentified endocrine disorders were recognized and addressed with the endocrinologist. This recognition occurred in the clinic setting with patients, as well as in the weekly division meetings and bi-monthly patient care rounds with providers. The presence of an endocrinologist promoted awareness of previously unaddressed endocrine disorders in our pediatric CF population.

### Umass CF/Endocrinology clinic model

The CF/Endocrinology clinic was established as a half-day clinic every Thursday afternoon. Initially, all patients, regardless of age, who were scheduled to see their pulmonologist, were automatically referred to the pediatric endocrinologist for assessment during the same clinic session. If there were no endocrine concerns, the patient was scheduled to see the endocrinologist annually for re-evaluation and to undergo an annual oral glucose tolerance test (OGTT), when appropriate. However, if an endocrine concern was identified during the introductory meeting, initial assessment, weekly division meetings or bi-monthly patient care rounds, the patient was seen more frequently. Endocrine concerns included, but were not limited to, disorders of linear growth, failure to thrive/poor weight gain (secondary to dysglycemia, thyroid disease, adrenal insufficiency, or constitutional delay), disorders of puberty, menstrual irregularities, vitamin D deficiency, bone disease, hypogonadism, adrenal insufficiency, glucose dysregulation, and CFRD. The pediatric endocrinologist was also tasked with ensuring that patients completed necessary endocrine-related testing, such as bone density (DEXA) scans and annual OGTTs. Abnormal OGTTs requiring further education and/or treatment were bridged by the pediatric endocrinologist to the UMass diabetes team, consisting of diabetes nurses and educators, as well as a diabetes psychologist, nutritionist and social worker.

### Data analysis

This study was a retrospective review of the UMass electronic medical records to assess changes after establishing the CF/Endocrinology Clinic, which included an automatic endocrinology referral system. All data was determined from an EMR chart review. The study protocol was approved by our Institutional Review Board, and the need for patient consent was waived because of the study design. We compared the rate of CF referrals to pediatric endocrinology, as well as the rate of OGTT and DEXA screening tests, before and after integration of a pediatric endocrinologist into the pediatric CF clinic in March 2017. GraphPad statistical software was used and Fishers Exact Test statistical analysis was completed. The pre-integration time period extended from 2013 to 2016 and the post-integration period extended from 2017 to 2020. In addition, we recorded the types of endocrine disorders that were diagnosed and/or managed in the post-integration period.

## Results

The percentage of CF pediatric patients in our clinic referred to pediatric endocrinology increased from 4% prior to integration of a pediatric endocrinologist in the clinic to 82% after integration (p < 0.0001). The remainder of the 18% of patients were unable to attend the combined CF/endocrinology clinic due to scheduling restraints. Additionally, OGTT and DEXA scan screening rates increased from 7% to 65% (p < 0.0001) and from 6% to 63% (p = 0.0011), respectively (Table 1)

**Table 1:** Endocrine-related patient care before and after integration of a pediatric endocrinologist in the CF clinic.

	Pre-Integration Period(2013–2016)	Post-Integration Period(2017–2020)	P value
Pediatric endocrinology referral	4% *(2/47)*	82% *(45/55)*	P < 0.0001
Annual OGTT screening rate of those who met criteria	7% *(3/41)*	65% *(30/46)*	P < 0.0001
DEXA scan screening rateof those who met criteria	6% *(1/16)*	63% *(12/19)*	P = 0.0011
Endocrine disorders evaluated	Predominantly CFRD	See Table 2	

CFRD, cystic-fibrosis related diabetes; DEXA, bone density; OGTT, oral glucose tolerance test.

Prior to integration of a pediatric endocrinologist in our multidisciplinary CF clinic, most pediatric CF patients were evaluated by pediatric endocrinology only for clinically overt CFRD. After integration, the diversity of endocrine disorders being evaluated and managed increased substantially. Table 2 shows the percentage of the UMass pediatric CF population currently under evaluation and/or management for a wide variety of endocrine conditions, with vitamin D insufficiency/deficiency (37.2% of clinic patients), glycemic dysregulation (36.8%), and failure to thrive/poor weight gain (17.5%) being the most common.

**Table 2:** Conditions currently under evaluation and/or management at the CF/Endocrinology clinic.

Condition	Percentage of Patients^a^
Disorders of linear growth	15.7
Failure to thrive/poor weight gain	17.5
Disorders of puberty	12.8
Menstrual irregularities	11.1
Vitamin D insufficiency/deficiency	37.2
Bone disease	15.8
Hypogonadism	10.3
Adrenal insufficiency	0.2
Glucose dysregulation	36.8
Cystic-fibrosis related diabetes	8.7
Endocrine disorders unrelated to cystic fibrosis	7.0

a Percentages of all clinic patients in December 2020.

## Discussion

Lack of a multidisciplinary approach to patient care in the outpatient setting is disadvantageous for individuals with a chronic systemic disease [3]. Delivering optimal care is the goal of any medical center, and a multidisciplinary clinic provides the environment necessary to address comorbidities that may otherwise be overlooked [24]. Our study demonstrated that implementation of a collaborative Pediatric CF/Endocrine Clinic with automatic referrals to pediatric endocrinology increased identification of endocrine disorders and increased OGTT and DEXA rates.

Many obstacles, including time, resource, and space constraints, limit the ability of many health centers to implement a multidisciplinary model of care. A lack of shared physical space, billing and documentation impediments that inadequately reflect the added value of a collaborative approach, insufficient understanding of the benefits of a multidisciplinary approach by hospital systems and insurance companies of the cost-effective and improved care provided to patients, and lack of disease-specific resources (such as staff and equipment) all act as barriers to establishing a multidisciplinary clinic [3]. Automatic referrals likely ensure patient volumes sufficient enough to justify the added expense of an additional sub-specialist in clinic.

However, once these impediments are overcome, the benefits of a “one-stop” team approach to systemic disease are numerous. The efficiency of combining care in one location benefits both patients and providers and promotes increased communication and cooperation amongst the health care team and with the patient. When multiple specialists approach treatment decisions together with the patient in a combined clinic setting, patent satisfaction and outcomes are superior [4], [5], [6], [7], [8], [9], [10], [11], [12], [13], [14]. In addition to these advantages, a multidisciplinary clinic provides an ideal opportunity to conduct observational studies that may overlap between the subspecialties involved [24].

In today’s healthcare system, the cost of care is important to consider. Although our study does not specifically analyze the cost effectiveness of the multidisciplinary care model, direct communication between subspecialty providers allowed for the ordering of appropriate tests without repeating or performing unnecessary studies. Additionally, earlier identification of CF endocrine co-morbidities is expected to reduce costs of overall care. The health and cost benefits of the multidisciplinary model likely outweigh the increased cost and clinic time of an additional subspecialty visit, as follow-up is determined on the presence and severity of endocrine disease. Since initiation of the CF/endocrinology clinic, no reimbursement challenges have been identified.

Our study showed significant statistical and clinical improvements in screening rates for several endocrine co-morbidities in pediatric CF patients, including bone disease and CFRD, after integration of a pediatric endocrinologist into our clinic, with automatic referrals to endocrinology. Many patients were previously unaware of their comorbid endocrine disorders and expressed satisfaction that the multidisciplinary approach led to the diagnosis and treatment of these conditions, which often improved their pulmonary CF symptoms as well.

Limitations of this study include analysis of a single center study with a small number of patients assessed during limited time periods. Additionally, our study does not assess time to referral, or data on patient satisfaction or the cost effectiveness of a multidisciplinary clinic. However, although this study has limitations, it provides a blueprint for an integrated clinical model and presents early data supporting the benefits of this approach. Healthcare providers, payors and administrators are encouraged to investigate further ways to adopt multidisciplinary care models in clinics managing complex diseases. Subsequent improvements in patient morbidity and mortality justify the time and coordination required to establish these care models.

## Funding

This research did not receive any specific grant from funding agencies in the public, commercial, or not-for-profit sectors.
